# Comprehensive review for healthcare data quality challenges in blockchain technology

**DOI:** 10.3389/fdata.2023.1173620

**Published:** 2023-05-12

**Authors:** Ahmed AbuHalimeh, Omar Ali

**Affiliations:** ^1^Donaghey College of Science, Technology, Engineering, and Mathematics, University of Arkansas, Little Rock, AR, United States; ^2^College of Business Administration, American University of the Middle East, Kuwait City, Kuwait

**Keywords:** blockchain technology, data quality, information quality, challenges, healthcare

## Abstract

There are several features inherent in blockchain, including decentralized storage, distributed ledger, immutability, security and authentication, and it has shifted away from the hype to be used practically in different industries, such as in the healthcare sector. The use of blockchain technology has allowed the provision of improved services to industries. The objective of this paper is to demonstrate how the use of blockchain is influenced by data quality issues in the healthcare industry. The article is structured as a systematic literature review study that uses several articles issued in various databases from 2016 onwards. In this review study, 65 articles were chosen and grouped into a single key aspect of the challenge in the healthcare sector. The findings obtained were analyzed based on factors in three domains, classified as issues pertinent to the adoption, operational and technological domains. This review study aims to use the findings to provide support to the practitioners, stakeholders and professionals, whose purpose is to carry out and manage transformation projects pertinent to blockchain in the field of healthcare. In addition, the organizations would be facilitated in their decision-making processes when the potential blockchain users are made to comprehend the implicit factors related to blockchain.

## Introduction

The rapid uptake of digitization in healthcare has led to the generation of massive electronic records about patients. Such growth poses unprecedented demands for healthcare data protection while in use and exchange (Ali et al., 2021). The rise of blockchain technology as a responsible and transparent mechanism to store and distribute data is paving the way for new potentials of solving serious data privacy, security, and integrity issues in healthcare. Blockchain technology has attracted considerable attention from industry as well as academics over the past few years. Indeed, new blockchain applications and research studies surface every day (Katuwal et al., 2018; Prokofieva and Miah, 2019; Ali et al., 2021; Mamun, 2022). A blockchain technology is identified as a distributed ledger technology for peer-to-peer (P2P) network digital data transactions that may be publicly or privately distributed to all users, allowing any type of data to be stored in a reliable and verifiable way (Zhang et al., 2018; Ali et al., 2021).

The focus of the blockchain, which is a technology that was developed recently, is on the models and inventions of the Internet of Things (IoTs) and the evolution of artificial intelligence (AI) (Ali et al., 2021). The field of healthcare constitutes a traditional industry; it is quite rigid in measurement because of the aspects of change and resistance to innovative procedures (Ali et al., 2021). In the past few years, attention is being given to various issues in the field of healthcare (for example, information security, and privacy) all over the world (Mamun, 2022). It is increasingly acknowledged that blockchain technologies are a means of addressing the information available regarding distribution issues. It was demonstrated in the latest report by Market Research Future (Prokofieva and Miah, 2019) that it was expected that by 2023, blockchain technology in healthcare would create a value of more than 42 million and attain a compound yearly growth rate of 71.8%.

It is predicted that the worldwide blockchain AI market would increase at a significant speed from 2020 to 2027 (Prokofieva and Miah, 2019). It is expected that this significant growth in blockchain technology would give rise to higher transparency, greater traceability, better privacy and security, improved efficiency as well as lower costs (Prokofieva and Miah, 2019). All industries are likely to be affected by blockchain technology, which would help in generating opportunities for enhancing business procedures and developing trust in data sharing and records management throughout the board. Security and trust are guaranteed when carrying out transactions through this technology (Katuwal et al., 2018; Zhang et al., 2018). Though there is increasing hype about blockchain technology that offers various advantages like privacy, security and transparency of operations, leading to more rapid transaction processing and data exchange, it is still considered by the healthcare industry as a distracting technology that one cannot trust and which requires management because of certain data quality issues.

Critical issues are being experienced by the healthcare industry's data management systems in the present times in the areas of traceability, data transparency, audit, immutability, flexible access, data provenance, security, privacy and trust (Abu-Elezz et al., 2020; Yaqoob et al., 2021). For instance, a Global Blockchain Survey was carried out by Deloitte at the beginning of 2019. It was claimed by 29% of the participants of this study that one of the main obstacles to the adoption of blockchain technology is an inadequate understanding of this technology (Katuwal et al., 2018). This is a significant quality issue pertinent to blockchain technology (Griggs et al., 2018; Prokofieva and Miah, 2019). Various other surveys simultaneously stress the fact that the understanding of blockchain technology in general and its use in healthcare in specific is only in its initial phase (Prokofieva and Miah, 2019). Very few studies have been carried out, specifically systematic literature review (SLR), which deal with the application, opportunities and challenges of blockchain in the field of healthcare because there has been limited research on blockchain and healthcare (Prokofieva and Miah, 2019).

There are limited robust, SLR about the existing blockchain-enabled state-of-the-art applications in the literature. The studies available do not address the various applications of blockchain in healthcare in its entirety. The focus is mostly on presenting a description of blockchain and briefly explaining how it is used. Blockchain technology has several applications and functions in the healthcare industry. By facilitating the safe transfer of patient medical records, controlling the drug supply chain, and facilitating the safe transfer of patient medical records, ledger technology aids in the discovery of genetic code by medical researchers. Some amazing and technically advanced aspects used to create and apply blockchain technology include protection of healthcare data, various genomics management strategies, electronic data management, medical records, interoperability, digitalized tracking, issue outbreaks, etc. The main drivers behind the growth of blockchain technology are its entirely digital components and applications in the healthcare industry. In a few studies, the existing blockchain research and its practical applications are explained. In addition, blockchain applications are reviewed and technical issues are elaborated, as well as the latest developments in addressing the challenges and the future path that would be taken by this technology. It is asserted by the researchers that further studies need to be carried out for obtaining a better understanding, characterization and assessment of the efficacy of blockchain technology in the field of healthcare (Zhao et al., 2016; Tama et al., 2017; Dimitrov, 2019; Abu-Elezz et al., 2020; Yaqoob et al., 2021).

However, previous studies have made little attempt to comprehensively summarize the existing knowledge by using SLRs (Angraal et al., 2017). For example, bibliometric techniques were used by Hölbl et al. (2018) to provide a summary of blockchain research patterns and components related to the implementation of blockchain in the field of healthcare. In Angraal et al. (2017), the different blockchain platforms have been developed to deploy blockchain in healthcare. Also, another study by Agbo et al. (2019) addressed different examples of the implementation in the healthcare of blockchain technology, the problems, and their potential solutions. In diverse contexts where this technology was implemented a research by O'donoghue et al. (2019) addressed design choices and tradeoffs made by the researchers. The research studies by Abou Jaoude and Saade (2019) and Hasselgren et al. (2020) have discussed the Blockchain-based applications throughout numerous industries and addressed many contexts of use for this technology in a broad manner. Recently Hasselgren et al. (2020) reviewed 39 studies to present an overview on common channels and other areas where blockchain technology is utilized for healthcare enhancement. Although these systematic literature reviews have a contribution to the extent of knowledge, their emphasis has been mainly on synthesizing or delineating blockchain technology patterns and areas (Hölbl et al., 2018; Agbo et al., 2019; Hasselgren et al., 2020). However, researchers will get benefit from a concentrated discussion on the implications of its adoption (Risius, 2017), along with concrete obstacles and areas for progress for advancing the field, due to the reach and diversity of previous blockchain studies (Agbo et al., 2019). Through assimilating existing information and describing focus areas that require considerable academic attention, review based research will assist in meeting these needs (Gopalakrishnan, 2013; Aznoli, 2017; Agbo et al., 2019). As a result of this necessity, we perform an SLR on the healthcare data quality challenges in blockchain technology application. This SLR presents a valuable overview of ongoing research, gaps in current knowledge, and future avenues of research as well.

It is important to carry out additional research to support the current efforts being made to address various challenges faced during the application of blockchain technology, e.g., interoperability, privacy, scalability and security (Yue et al., 2016; Genestier et al., 2017; Kuo et al., 2019; Farouk et al., 2020; Yaqoob et al., 2021). There is an increasing need to expand knowledge so that an understanding of the uses and benefits of blockchain technology can be developed. Thus, a study needs to be carried out that focuses on the challenges of blockchain technology and its effect on the healthcare sector, which would give rise to various conventional issues concerning wasted resources, unfulfilled expectations and gradual implementation of emerging technologies (Prokofieva and Miah, 2019).

A SLR of blockchain technology was carried out in the field of healthcare to gain an improved understanding of the data quality changes of blockchain and its effect on the healthcare industry. The review aimed to identify the shortcomings associated with the data quality challenges and the future path concerning the application of blockchain in healthcare. Blockchain challenges are classified in this review as issues pertinent to adoption, operational and technological aspects.

“This review helps in determining healthcare data quality challenges concerning blockchain and presents a comprehensive review of the adoption and implementation of this technology in the field of healthcare”.

The organization of the rest of the paper is as follows: In Section Blockchain background, the background, definition, features and various categories of data quality of blockchain technology are presented. Section Research methodology elaborates on the SLR stages while Section Research discussion provides a discussion of the results. Section Research implications discusses the effects on theory and practice. In Section Research limitations and future directions, the study limitations are discussed as well as the future course of direction for research. Finally, in Section Conclusion, a conclusion is presented for this review study.

## Blockchain background

### Blockchain technology

Blockchain technology is a decentralized, distributed ledger that records transactions on multiple computers in a secure, transparent, and tamper-proof way (Abou Jaoude and Saade, 2019; Agbo et al., 2019; O'donoghue et al., 2019). The technology was originally created to support the digital currency, Bitcoin, but has since expanded to support a wide range of applications across industries (Mendling et al., 2018). At its core, a blockchain is a series of blocks that contain information about transactions. Each block is linked to the previous one in the chain, forming a continuous and unbroken chain of data. Once a block is added to the blockchain, it cannot be altered or deleted without consensus from the network, making it an immutable record of all transactions (Kuo et al., 2019; Hasselgren et al., 2020).

One of the key features of blockchain technology is its decentralization (Angraal et al., 2017). Rather than relying on a central authority to validate transactions, blockchain technology uses a network of computers to validate and verify transactions (Yli-Huumo et al., 2016). This creates a more secure and transparent system that is less vulnerable to fraud, corruption, or other forms of manipulation. Another important feature of blockchain technology is its transparency (Silva et al., 2019). Since all transactions are recorded on the blockchain, anyone can view them, making it easier to track and trace transactions. This transparency can be particularly valuable in industries such as healthcare, where it is important to ensure that data is accurate and trustworthy (Yli-Huumo et al., 2016). In addition to decentralization and transparency, blockchain technology also offers a high degree of security. Each block in the blockchain is secured through a cryptographic hash function, which ensures that the data cannot be altered without being detected. This makes it difficult for hackers or other malicious actors to tamper with the data on the blockchain.

There are also different types of blockchains, including public and private blockchains (Agbo et al., 2019). Public blockchains are open to anyone (Efanov, 2018), while private blockchains are restricted to specific users or organizations (Angelis and da Silva, 2019). Hybrid blockchains combine elements of both public and private blockchains (Yli-Huumo et al., 2016). Overall, blockchain technology has the potential to transform a wide range of industries, including healthcare, finance, supply chain management, and more. However, there are still challenges to widespread adoption, including issues around scalability, interoperability, and regulatory compliance (Tama et al., 2017; Abu-Elezz et al., 2020; Yaqoob et al., 2021). As the technology continues to evolve and mature, it is likely that we will see even more innovative applications of blockchain technology in the years to come (Kuo and Kim, 2017).

Blockchain refers to the decentralized manner in which validation and tamper-free transactions that are constant over a large number of participants are addressed, also referred to as nodes (Glaser, 2017; Müller-Bloch and King, 2018). It is asserted in a study carried out by Müller-Bloch and King (2018), blockchain may be considered a kind of distributed ledger technology that offers confidence to the user that there has been no tampering with the information that has been archived, for example, certificates. It has been demonstrated in different studies (Nærland et al., 2017) that blockchain has the potential to reduce transactional vagueness, insecure states and uncertainty by providing full disclosure of transactions, along with homogenous and substantiated facts to all participants in the network.

### Blockchain characteristics

Blockchain technology possesses a number of characteristics, including immutability, shared and public access, low friction, peer verification, cryptography immutability, decentralization, versatility, and automation. When assessing blockchain technology in healthcare, trust and decentralization are the two main elements that need to be identified because both are significant features in the adoption of blockchain technology in the healthcare sector, which may function as a unique solution for issues prevalent in this industry (Seebacher and Schüritz, 2017). Trust and decentralization in blockchain technology will be explained in more detail in the subsequent sections.

#### Trust

This is the most important feature of blockchain technology that is concealed by the decentralized mechanism of the technology. There is a proof-of-work protocol that secures the network and eliminates the need to include any third party to validate and record transactions. Through this protocol, the users of blockchain technology do not have to depend on third parties to secure their assets and transactions. Blockchain technology allows its members to create a shared and safe network.

The relationship was revealed to the public. Participants have full disclosure on the system's activities because there is a shared view on all passed and current transactions. Because there is no single intermediary controlling the system, users can interact directly, resulting in less friction. Trust can be also fostered by the technology's inherent feature of ensuring the integrity of data stored in the database itself, and through the database's immutable design, which means that once a transaction is added to a block, which is then added to the blockchain, it cannot be changed. This process is aided by the use of a mechanism known as the consensus mechanism (Seebacher and Schüritz, 2017).

#### Decentralization

Decentralization is one of the most significant features of blockchain technology (Seebacher and Schüritz, 2017) and its most important aspects are resistance to censorship and immutability. Certain significant terminologies can be used to express the vital characteristics of decentralization that are part of blockchain technology, such as pseudonyms of members (Zyskind and Nathan, 2015), the prospective usability of automation (Guo and Liang, 2016; Xu, 2016), data redundancies (Hull et al., 2016), and involvement of peers in development “versatility” (Zhao et al., 2016). Trust and decentralization are both significant features in the adoption of blockchain technology in the healthcare sector, which may function as a unique solution for issues prevalent in this industry. There is a lot of importance of trust in the healthcare industry, for example, the medical data of the patient are typically distributed over different facilities, insurance providers and medical care centers (Yaqoob et al., 2021). These different aspects of a patient's data need to be combined in an automated manner so that an accurate medical history of the patient can be obtained. For this, all of the patient's medical data (such as symptom information, prescriptions, treatment approach, etc.) can be stored on a blockchain that always stores updated, tamper-free and traceable records (Yaqoob et al., 2021). Healthcare professionals thus trust the data they are using and offer timely, efficient and appropriate treatments to patients. The technology enters into several aspects of healthcare because of its broader applicability. Apart from handling validation and tamperproof transactions with uniformity across a large number of participants, blockchain technology also securely stores information and allows the incorporation of automation into electronic medical records (EMRs). The technology is also capable of bringing substantial changes in the sharing of health information and ensuring higher data privacy, improved patient treatment, better healthcare quality and the use of more extensive medical science. As blockchains are more challenging than they appear to be, there are a few vital concerns that need to be taken into account before considering an extensive rollout as being secure and efficient (Singh et al., 2022). There are increasing demands of the healthcare sector on blockchain developments, and it was demonstrated in a recent study by Deloitte that new possibilities for using blockchain are actively being sought by the traditional industry to fulfill its significant requirements (Deloitte, 2018). Just like with any new technology, blockchain should be examined by healthcare organizations in terms of their particular needs to offer clinicians with the expertise they need to effectively use these resources.

### Blockchain categories

There are three major categories into which blockchain is grouped, i.e., public, private and consortium (Ali et al., 2021). An identical group of rights and prerogatives is held by every participant in the public blockchain. This involves giving equal authority to every participant instead of giving centralized authorization to a third party (Xu et al., 2016; Sankar et al., 2017). Every party is also free to join or leave the network. For each participant, this feature is free of cost, and any source can verify the transactions. The verification of transactions in consortium blockchain cannot be carried out by anyone. This also suggests that there are very few vital members that are authorized to substantiate the transactions. Some members have the option of verifying their transactions before validation is carried out; these critical members need to have access to the agreement. Centralized configuration protocols are also guaranteed for private blockchains.

## Healthcare data quality

The American Institute for Health Management (The American Institute for Health Care Management, 2022) stated that implementing Information Technology (IT) solutions that help in enhancing the quality of their care data is the challenge faced by healthcare organizations in the present times. The data and information in this industry essentially need to be of high quality. The innate problems related to poor-quality source data and data collection or entry errors cannot be solved by even the most advanced information systems (ISs) (The American Institute for Health Care Management, 2022). The features can serve as valuable tools to develop techniques that ensure that the healthcare data is of high quality (The American Institute for Health Care Management, 2022). Often, data quality is described as the suitability of data for use and hence, various data quality dimensions determine data quality, with their relevance depending on the context of the application. Being a multi-faceted concept, there are various characteristics of data quality, for example, validity, reliability, accuracy, consistency and accessibility, among others (Halimeh, 2011; Data Quality's Importance to Blockchain, 2022). From these, accuracy may be described as appropriately representing the practical world phenomenon that it intends to model (Halimeh, 2011). It is ensured by validity and accuracy that the original data source is not misrepresentative or corrupt. In addition, consistency and reliability ensure that a fixed standard is followed by the information all through its life cycle and across the organization (Halimeh, 2011). Accessibility makes certain that authorized individuals have access to the data when and wherever required (Halimeh, 2011), for example, medical or health records. For various healthcare institutes, high-quality data is an objective, particularly in those that depend on data-based decisions and carry out tasks involving data analytics, such as AI-powered or machine-learning applications.

Healthcare data quality typically implies the level of confidence of the user in the data. When the standards given below are maintained, this degree of confidence is the highest (The American Institute for Health Care Management, 2022): Quality data needs to be sourced by healthcare organizations, along with developing robust procedures so that it can be managed in the long run in a conceptually organized way. This would allow them to increase the speed of their current procedures as well as create learning opportunities that help in making smarter policy decisions that can have an impact on all stakeholders (The American Institute for Health Care Management, 2022). In addition, with time, clean health records and downstream datasets can increase in value. This happens when parties across and between organizations start sharing more data and hence obtain greater insight into their systems than they otherwise could have if their data was stored separately as in the past (The American Institute for Health Care Management, 2022).

### Data quality in blockchain

There is remarkable potential for data quality standards and blockchain technology as a means of enhancing healthcare data quality. The legibility and accessibility of healthcare data and information are enhanced by Electronic Medical Records (EMRs) (Data Quality's Importance to Blockchain, 2022). Data quality and blockchain technology have a powerful relationship, where blockchain technology should bring about a significant improvement in data quality. The two main aspects (characteristics) of data quality that are most appropriate to blockchain are consistency and integrity (Data Quality's Importance to Blockchain, 2022). The similarity of other sources of data that refer to the same concept is measured through the equivalence or redundancy of distributed data. Dan Mayer (Data Quality's Importance to Blockchain, 2022) stated that several advantages are offered by blockchain that are consistent with data quality enhancements, for example, the ability to offer complete audit trails of transactions and authenticate entities.

The objective of blockchain technology is to bring significant improvements in data quality. There are various data quality problems and difficulties that need to be taken into account even though this technology offers various data quality advantages; for example, the ability to offer complete audit trails of transactions and authenticate entities (Data Quality's Importance to Blockchain, 2022).

For instance, data integrity is likely to give rise to a few issues. Weak data may be added to a system that does not provide an accurate representation of the actual world. Ensuring the precision of healthcare data is another issue, which may occur due to price discrimination, human and administrative errors, insurance market competition, and not giving correct data to avoid tax. There are inaccuracies in the majority of the prevailing healthcare data registries because of different reasons. Hence, it is important to clean the healthcare data registers and update them before using blockchains to store data (Yaqoob et al., 2021). There are several advantages of a blockchain, particularly when tragedies due to natural calamities occur, where the traditional healthcare system experiences a loss of sensitive health data. In blockchain technology, healthcare data is stored on a distributed platform that is safe from physical damage.

A significant part is played by data consistency in allowing the various blockchain networks to interact. When there are variations in various consensus models, transaction methods, and smart contract functionalities (Yaqoob et al., 2021), data inconsistencies can emerge. This review study will present various other healthcare data quality issues that will help professionals, stakeholders as well as practitioners to execute and manage transformation projects pertinent to blockchain in the field of healthcare. In addition, the decision-making processes of the organizations would be facilitated when the prospective blockchain users comprehend the implicit factors that are related to blockchain.

## Research methodology

The existing review method takes guidance from the study of reference (Ali et al., 2018). The sequence of this review follows a particular process and protocol that involves identifying, choosing, and evaluating the literature based on the parameter of relevance. The purpose of the article is to improve the efficiency of the review process (Tranfield et al., 2003); and to make it objective, replicable, candid, impartial, and meticulous (Boell and Cecez-Kecmanovic, 2015). Based on the studies of reference (Keele, 2007; Ali et al., 2020, 2021), the review is organized as a three-phase process that involves planning, executing, and reporting. These processes will be explained subsequently.

### Planning stage

In this stage, the review requirements are identified. Though there are studies that examine the healthcare data quality issues experienced in blockchain technology, there is inadequate academic literature on this topic and its review. Thus, the paper will offer an extensive analysis of the knowledge that is available in research and practice. The research questions were also determined in the planning stage, the fundamental one being: What are the main healthcare data quality challenges experienced in blockchain technology? Therefore, the purpose of the review is to specifically answer this research question so that future researchers can acknowledge the issues involved in healthcare data quality for the adoption of blockchain technology in the future. The criterion for choosing the article using particular methods and approaches was also established in this stage. An integrated search strategy was included in this stage to incorporate a search engine, i.e., an automated search, on various electronic databases as well as a manual review of different publications (Golder et al., 2014). The online databases selected in the present review comprise Scopus, ACM digital library, Emerald, Science Direct, Web of Science, and IEEE. Tools were also strategically filtered so that the required search results for each of the selected databases could be limited (McLean and Antony, 2014). The extensive manual review involved reading the title and abstract of every research article (Kassab et al., 2019), after which the assorted content of these articles was systematically read to eliminate the irrelevant ones (Ali et al., 2018). After this, the research review protocol was developed, based on which the existing theoretical and practical viewpoints on the theme were comprehended. The preliminary classification framework presented by Ngai and Wat was a part of this review (McLean and Antony, 2014). The suggested cataloging framework is included in the study, which involves three different types that are linked to different elements of the key challenges associated with healthcare data in terms of using blockchain technology. These are adoption, technological and operational challenges. Apart from these, it includes the subcategories in each component of the framework (refer to **Table 2**).

### Execution stage

The approaches of the planning stage were extended in this stage to obtain the articles that were relevant to this review paper. The review study used the following key approaches: (1) determining the search terms and words as a continuously evolving process that starts with the use of distinct, technical terms pertinent to the field (Ngai and Wat, 2002; Hu and Bai, 2014). The following keywords were determined in this study: “issues” OR “challenges” OR “obstacles” OR “barriers” OR “Data” AND “blockchain” AND “quality”. (2) To improve the appropriateness of the search results, database filtering tools were employed; the temporal restriction feature was implemented so that the search period could be restricted to between 2012 and 2022 (Zhang et al., 2014). (3) Manually checking of the results was then carried out to ensure that they were relevant, with the focus being on title and abstract (Pucher et al., 2013). (4) After this, comprehensive analysis of the shortlisted articles from the preceding step was carried out to achieve appropriate knowledge, theory, and information on the field of research (Shea et al., 2007). (5) Lastly, quality assessment criterion was implemented to confirm that all articles filtered systematically by following the previous steps were relevant to the study domain (Hu and Bai, 2014). The checklist given in the studies by reference (Ali et al., 2018; Sadoughi et al., 2020) were used to approve the shortlisted articles. The checklist comprised the following: the research objectives are explained clearly, an appropriate description of the data employed is provided, a comprehensive explanation of the methodology used is provided, and the research findings are explained systematically so that the research questions can be answered. Table 1 presents details on the study selection process and their corresponding findings.

**Table 1:** Selection process and result.

**Stage**	**Result**
**Study selection process and result**	
Stage 1: Search based on the key words	317
Stage 2: Database filtering tools applied	198
Stage 3: Eliminate articles based on title and abstract	127
Stage 4: Eliminate articles based on full text scanning	86
Stage 5: Eliminate articles based on quality assessment	49
Total number of articles that been used in this review study	49

### Summarizing stage

The existing review was carried out from January 24th, 2022 to April 23rd, 2022, taking into account the protocols shown in the planning stage. A total of 317 articles were identified in the preliminary search. In the end, only 49 articles were identified after being filtered through the review stages, quality assessment criterion and categorization model presented, as shown subsequently (Table 2).

**Table 2:** Categorization framework dimension category type.

**Dimension**	**Category**	**Type**	**References**
Healthcare data challenges	Technological	Security	Golder et al., 2014; Randall et al., 2017; Tama et al., 2017; Abou Jaoude and Saade, 2019; Agbo et al., 2019; Alonso et al., 2019; Beinke et al., 2019; Chen et al., 2019; Clim et al., 2019; Dimitrov, 2019; Khezr et al., 2019; McGhin et al., 2019; Prokofieva and Miah, 2019; Shahnaz et al., 2019; Shuaib et al., 2019; Siyal et al., 2019; Yaqoob et al., 2019; Zubaydi et al., 2019; Maesa and Mori, 2020; Pandey and Litoriya, 2020; Tanwar et al., 2020; Adere, 2022; Attaran, 2022; Saeed et al., 2022
		Privacy	Angraal et al., 2017; Liang et al., 2017; Sharma, 2018; Zhang et al., 2018; Abou Jaoude and Saade, 2019; Agbo et al., 2019; Alonso et al., 2019; Beinke et al., 2019; Casino et al., 2019; Clim et al., 2019; Esmaeilzadeh and Mirzaei, 2019; Khezr et al., 2019; McGhin et al., 2019; Prokofieva and Miah, 2019; Shuaib et al., 2019; Siyal et al., 2019; Yaqoob et al., 2019; Khan et al., 2020; Maesa and Mori, 2020; Pandey and Litoriya, 2020; Adere, 2022; Attaran, 2022; Saeed et al., 2022
		Integration	Golder et al., 2014; Guo and Liang, 2016; Hull et al., 2016; Angraal et al., 2017; Tama et al., 2017; Griggs et al., 2018; Müller-Bloch and King, 2018
	Adoption	Interoperability	Tama et al., 2017; Beinke et al., 2019; Cao et al., 2019; Abu-Elezz et al., 2020; Tanwar et al., 2020; Yaqoob et al., 2021; Attaran, 2022
		Compatibility	Liang et al., 2017; Randall et al., 2017; Katuwal et al., 2018; Abou Jaoude and Saade, 2019; Alonso et al., 2019; Kassab et al., 2019; McGhin et al., 2019; Yaqoob et al., 2021; Attaran, 2022
		Standardization	Randall et al., 2017; Katuwal et al., 2018; Abou Jaoude and Saade, 2019; McGhin et al., 2019; Attaran, 2022
		Data governance	Angraal et al., 2017; Chen and Huang, 2018; Gökalp et al., 2018; Cao et al., 2019; Khezr et al., 2019; Patel, 2019; Abu-Elezz et al., 2020; Attaran, 2022
	Operational	Scalability	Hussein et al., 2018; Katuwal et al., 2018; Agbo et al., 2019; Casino et al., 2019; Clim et al., 2019; Kassab et al., 2019; Khezr et al., 2019; McGhin et al., 2019; Shuaib et al., 2019; Maesa and Mori, 2020; Sengupta et al., 2020; Adere, 2022; Mamun, 2022
		Data availability	Wood et al., 2016; Randall et al., 2017; Katuwal et al., 2018
		Accessibility	Angraal et al., 2017; Genestier et al., 2017; Farouk et al., 2020; Yaqoob et al., 2021
		Usability	Adere, 2022; Saeed et al., 2022
		Data sharing	Yue et al., 2016; Khezr et al., 2019
		Data processing speed	Vora et al., 2018; Beinke et al., 2019; Esmaeilzadeh and Mirzaei, 2019; Ornes, 2019; Zheng et al., 2019; Abu-Elezz et al., 2020; Maesa and Mori, 2020; Pandey and Litoriya, 2020

These were appropriate for significant challenges of healthcare data quality by employing blockchain technology; pertinent to adoption, operational and technological challenges that stem from the review process.

## Research discussion

The healthcare data quality issues inherent in blockchain technology are presented and discussed in this section. There are significant challenges for data quality in healthcare, and to make informed clinical decisions, service providers and patients should have integrated security as well as trusted data. There are various features and functions of blockchain technology that may be used in the field of healthcare. Various medical and healthcare systems can use these functions. Blockchain offers various advantages, e.g., trust, transaction transparency, and security, which supposedly lead to more rapid transaction processing and exchange of data (Watson, 2015; Abou Jaoude and Saade, 2019). However, before adopting and implementing blockchain technology in the field of healthcare, various issues need to be taken into account that may have an impact on the quality of data being used and shared, such as accuracy, consistency, and accessibility of prescriptions history, treatment approach, patient records, and symptom data.

### Technological challenges

While blockchain technology is designed to be secure, there are still concerns around data privacy and security. Healthcare data is particularly sensitive and must be protected from unauthorized access or disclosure. There is also the risk of data breaches, which can compromise patient privacy and put them at risk of identity theft or other forms of fraud. Also, healthcare organizations often use a variety of legacy systems that may not be compatible with blockchain, which can make implementation difficult and time-consuming.

Blockchain technology has the potential to revolutionize the healthcare sector by enabling secure and decentralized storage and sharing of medical data (Abou Jaoude and Saade, 2019). However, there are several data privacy and security challenges that need to be addressed before blockchain can be widely adopted in healthcare (Chen et al., 2019; Esmaeilzadeh and Mirzaei, 2019). Identity protection is one of the key challenges in adopting blockchain technology in healthcare is protecting patient identity (Casino et al., 2019). Healthcare data is often sensitive and must be protected from unauthorized access or disclosure (Zubaydi et al., 2019). With blockchain, data is stored in an encrypted form, but if a patient's identity is linked to their encrypted data, it could still be at risk of unauthorized access (Khan et al., 2020). Data breaches; while blockchain is designed to be secure, there is still a risk of data breaches. Hackers may target blockchain systems to gain access to sensitive healthcare data, which could result in identity theft, fraud, or other forms of harm (Agbo et al., 2019; Khan et al., 2020). Data integrity is another challenge is ensuring the integrity of healthcare data on the blockchain. Healthcare data must be accurate and up-to-date, and there is a risk of errors or tampering with the data, which could have serious consequences for patient care (Randall et al., 2017; Agbo et al., 2019; Chen et al., 2019; McGhin et al., 2019). Regulatory compliance; healthcare is a heavily regulated industry, and there are strict regulations around the storage and sharing of medical data (Wood et al., 2016; Randall et al., 2017; Sharma, 2018; Alonso et al., 2019; Beinke et al., 2019; Clim et al., 2019; Khezr et al., 2019; McGhin et al., 2019; Prokofieva and Miah, 2019; Shahnaz et al., 2019; Shuaib et al., 2019; Siyal et al., 2019; Yaqoob et al., 2019; Zubaydi et al., 2019; Maesa and Mori, 2020; Pandey and Litoriya, 2020; Tanwar et al., 2020; Adere, 2022; Attaran, 2022; Saeed et al., 2022). Blockchain technology may not always meet these regulatory requirements, which can create barriers to adoption. Access control refers to controlling access to healthcare data on the blockchain can be challenging. Healthcare organizations must ensure that only authorized personnel have access to patient data, and that patients have control over who can access their data (Wood et al., 2016; Alonso et al., 2019; Beinke et al., 2019; Clim et al., 2019; Khezr et al., 2019; McGhin et al., 2019; Prokofieva and Miah, 2019; Shahnaz et al., 2019; Shuaib et al., 2019; Siyal et al., 2019; Yaqoob et al., 2019; Zubaydi et al., 2019; Maesa and Mori, 2020; Pandey and Litoriya, 2020; Tanwar et al., 2020; Adere, 2022; Attaran, 2022; Data Quality in Healthcare, 2022; Saeed et al., 2022).

Integration challenges are one of the major roadblocks to the adoption of blockchain technology in the healthcare sector (Tama et al., 2017; Griggs et al., 2018). Blockchain technology is a relatively new technology, and integrating it with existing healthcare systems can be a complex and time-consuming process. Some of the key integration challenges include. Legacy systems are related to healthcare organizations often use a variety of legacy systems, which may not be compatible with blockchain technology (Guo and Liang, 2016; Hull et al., 2016). Integrating blockchain technology with these legacy systems can be challenging and may require significant changes to the existing infrastructure (Angraal et al., 2017). Implementation costs is referring to implementing blockchain technology can be expensive, and healthcare organizations may not have the resources to invest in the technology. The costs associated with implementing blockchain technology, including hardware, software, and training, can be a significant barrier to adoption (Angraal et al., 2017; Seebacher and Schüritz, 2017). Resistance to change can be a major challenge in adopting blockchain technology in healthcare. Healthcare organizations may be reluctant to adopt new technologies, especially if they perceive them as risky or difficult to implement (Hull et al., 2016; Tama et al., 2017).

### Adoption challenges

This review study identified different blockchain adoption challenges in healthcare sector. Each of these challenges discussed next.

Interoperability is a significant challenge in adopting blockchain technology in the healthcare sector (Abu-Elezz et al., 2020; Yaqoob et al., 2021). Interoperability refers to the ability of different blockchain systems to communicate and exchange data with each other (Yaqoob et al., 2021). In healthcare, there are several different blockchain systems being developed, and it is important that they can all work together seamlessly to provide the maximum benefits to patients, healthcare providers, and other stakeholders (Cao et al., 2019). One of the key challenges to achieving interoperability in blockchain-based healthcare systems is the lack of standardization (Tama et al., 2017; Abu-Elezz et al., 2020; Tanwar et al., 2020; Yaqoob et al., 2021). There is currently no widely accepted standard for healthcare data exchange on blockchain platforms, which can make it difficult to integrate different systems. Without standardization, blockchain systems may use different data formats, terminologies, and other specifications that are not compatible with each other (Beinke et al., 2019; Attaran, 2022). This can create silos of data that cannot be shared, which can limit the effectiveness of blockchain technology in healthcare. Another challenge to achieving interoperability in blockchain-based healthcare systems is the need for secure data exchange (Beinke et al., 2019; Cao et al., 2019; Tanwar et al., 2020). Healthcare data is highly sensitive, and any exchange of data between different blockchain systems must be done securely to protect patient privacy. However, ensuring secure data exchange across different blockchain systems can be difficult, especially if they use different encryption and security protocols (Tanwar et al., 2020).

Standardization is a significant challenge in adopting blockchain technology in the healthcare sector (Katuwal et al., 2018; Abou Jaoude and Saade, 2019; Yaqoob et al., 2021). Standardization refers to the process of creating common technical and operational standards that enable different systems and stakeholders to work together seamlessly. Standardization is important because it ensures that data can be exchanged easily and accurately between different blockchain systems, which is essential in healthcare where patient data must be shared across different providers and organizations (Liang et al., 2017; Alonso et al., 2019; McGhin et al., 2019; Attaran, 2022). One of the key challenges to achieving standardization in blockchain-based healthcare systems is the lack of industry-wide standards (Katuwal et al., 2018; Yaqoob et al., 2021). Different blockchain systems may use different data formats, terminologies, and other specifications that are not compatible with each other, which can create silos of data that cannot be shared. This can limit the effectiveness of blockchain technology in healthcare, as it becomes difficult to exchange data between different systems. Another challenge to achieving standardization in blockchain-based healthcare systems is the need for regulatory guidance. Healthcare is a highly regulated industry, and regulatory bodies must provide clear guidance on the use of blockchain technology in healthcare to ensure that all stakeholders understand the legal and ethical implications of using the technology (Abou Jaoude and Saade, 2019; Alonso et al., 2019; Attaran, 2022). However, regulatory guidance around the use of blockchain technology in healthcare is still developing, which can create uncertainty around data privacy, security, and other issues (Kassab et al., 2019).

Data governance is a significant challenge in adopting blockchain technology in the healthcare sector (Data Quality's Importance to Blockchain, 2022). Data governance refers to the processes, policies, and standards that are used to manage the collection, use, sharing, and protection of data. In healthcare, data governance is particularly important because patient data is highly sensitive and must be protected to maintain patient privacy and comply with regulatory requirements (Liang et al., 2017). One of the key challenges to achieving effective data governance in blockchain-based healthcare systems is the need to balance patient privacy with the benefits of data sharing. Blockchain technology can enable secure, decentralized sharing of data, which can help to improve patient care and outcomes (Randall et al., 2017). However, this also raises concerns around patient privacy and the potential misuse of data. It is important to establish clear policies and standards around data sharing and use in blockchain-based healthcare systems to ensure that patient privacy is protected and that data is used ethically and responsibly (Kassab et al., 2019; Attaran, 2022). Another challenge to achieving effective data governance in blockchain-based healthcare systems is the need to manage data quality and accuracy (Liang et al., 2017; Randall et al., 2017; McGhin et al., 2019). Blockchain technology relies on accurate and high-quality data to function effectively, and any errors or inaccuracies in the data can compromise the integrity of the blockchain system. Healthcare data is often complex and heterogeneous, which can make it difficult to ensure data quality and accuracy. It is important to establish clear policies and standards around data quality and accuracy in blockchain-based healthcare systems to ensure that the data used is reliable and trustworthy (Liang et al., 2017).

### Operational challenges

The research determined that the scalability issue is caused by the various transactions and data that are permanently stored on each block. Scalability is a significant issue that can slow down the mainstream adoption of public Data Governance (Chen and Huang, 2018; Gökalp et al., 2018; Cao et al., 2019; Khezr et al., 2019; Patel, 2019; Yaqoob et al., 2019; Abu-Elezz et al., 2020; Attaran, 2022). Operational Scalability (Hussein et al., 2018; Katuwal et al., 2018; Agbo et al., 2019; Casino et al., 2019; Clim et al., 2019; Kassab et al., 2019; Khezr et al., 2019; McGhin et al., 2019; Shuaib et al., 2019; Maesa and Mori, 2020; Pandey and Litoriya, 2020; Sengupta et al., 2020; Adere, 2022; Mamun, 2022). Data Availability (Wood et al., 2016; Randall et al., 2017; Katuwal et al., 2018). Accessibility (Genestier et al., 2017; Farouk et al., 2020; Tanwar et al., 2020; Yaqoob et al., 2021). Usability (Adere, 2022; Saeed et al., 2022). Data Sharing (Yue et al., 2016; Khezr et al., 2019). Data Processing Speed (Vora et al., 2018; Beinke et al., 2019; Esmaeilzadeh and Mirzaei, 2019; Ornes, 2019; Zheng et al., 2019; Abu-Elezz et al., 2020; Maesa and Mori, 2020; Pandey and Litoriya, 2020). In the healthcare industry, blockchains determine how capable a system is of increasing or decreasing performance and cost following changes in application and system processing requirements. Nevertheless, there are a few blockchains, such as Ethereum, that are lagging extensively with respect to transaction speeds because they are only able to process around 20 transactions per second (Deloitte, 2018; Hussein et al., 2018; Katuwal et al., 2018; Agbo et al., 2019; Casino et al., 2019; Clim et al., 2019; Kassab et al., 2019; Khezr et al., 2019; McGhin et al., 2019; Shuaib et al., 2019; Abu-Elezz et al., 2020; Maesa and Mori, 2020; Sengupta et al., 2020; Adere, 2022; Mamun, 2022). The challenges presently faced by medical practitioners include the sharing and usability of healthcare data, where distinct information technology (IT) systems can communicate, exchange and utilize information (Adere, 2022; Saeed et al., 2022). For instance, it is essential to obtain the medical history of a patient before he/she is prescribed any medicine for appropriate treatment (Boell and Cecez-Kecmanovic, 2015). Another issue that is faced concerning the operational efficiency of blockchain is related to accessibility (Genestier et al., 2017; Farouk et al., 2020; Tanwar et al., 2020; Yaqoob et al., 2021). Blockchain suffers from a diversity problem just like any other disruptive technology, due to which accessibility is further restricted. Another significant challenge concerning communication is a lack of blockchain standards, because of inadequate interoperability (Tama et al., 2017; Beinke et al., 2019; Cao et al., 2019; Abu-Elezz et al., 2020; Tanwar et al., 2020; Yaqoob et al., 2021; Attaran, 2022). It is difficult to achieve appropriate data processing speed in public blockchains because to validate a transaction, several nodes need to attain a consensus. Each node should have access to the complete blockchain network to process transactions and attain a consensus (Vora et al., 2018; Beinke et al., 2019; Esmaeilzadeh and Mirzaei, 2019; Ornes, 2019; Zheng et al., 2019; Abu-Elezz et al., 2020; Maesa and Mori, 2020; Pandey and Litoriya, 2020). In public blockchains, this leads to latency problems. In contrast, giving access to the complete blockchain can give rise to privacy and security concerns (Yaqoob et al., 2021). Though blockchain technology has a huge potential and a lot of attention is being given to it, it was determined in the study that there is a powerful relationship between data quality and blockchain technology, where the technology should bring major improvements in data quality, but the way it affects healthcare data quality is in its initial stages. However, with the rapid development of the healthcare industry, it is expected that blockchain will soon have a significant positive impact on the healthcare sector. Blockchain technology is supposed to bring significant improvements in data quality, e.g., accuracy and security. Though several data quality advantages are provided by blockchain technology, e.g., the ability to offer complete audit trails of transactions and authenticating entities, various other data quality challenges exist that should be taken into account, for example, consistency, data integration, interoperability, scalability, security, and privacy, in addition to several others previously explained in this paper.

## Research implications

It is determined in this paper that there are significant theoretical contributions to information technology and systems in general and blockchain research in healthcare in specific. In addition, a significant classification framework pertinent to blockchain issues is presented in the paper. This paper presented particular categories and forms of challenges, i.e., technological, adoption, and operation, which showed the different issues that need to be studied in further detail in future blockchain-based application development and research. The classification framework will likely motivate and facilitate different research efforts from distinct fields of healthcare research, e.g., data quality, data security, health informatics, and healthcare applications, to bring about extensive involvement in the adoption and development of this technology. It is also likely that the suggested framework would guide blockchain researchers in their future endeavors. This framework may be employed by researchers in the field of information technology and information quality to comprehend their overall effect on quality. In addition, the framework offers generalization and flexibility of the categorization, which can help in considering and adopting new kinds of data quality issues that may emerge in future research.

## Research limitations and future directions

There are several limitations inherent in this study. Only journal articles, IEE, and conference proceedings included in Science Direction, Scopus, and Google Scholar sources were considered in the review. Blockchain is a comparatively new subject for the field of research and is not studied extensively; the study considered most of the papers issued since 2016. Because there are very few papers in the research field, issues are experienced in comparing and generalizing findings. Secondly, the focus of the study is only on the healthcare sector. The technology is still in its initial stages, and it is determined in the results of this research that additional studies need to be carried out immediately to evaluate blockchain technology. The issues pertinent to interoperability, security, scalability, and privacy continue to be the most evident difficulties faced during the implementation of blockchain in the healthcare industry (Abu-Elezz et al., 2020). Hence, various future research directions may be identified, as discussed subsequently. Additional studies need to be carried out to evaluate the security features of blockchain technology and its impact in such application environments that need a high degree of identity and security, similar to healthcare and government agencies (Katuwal et al., 2018; Abou Jaoude and Saade, 2019; Casino et al., 2019). Privacy and security risks may be experienced when access to the complete blockchain is provided. Another vital issue is determining how the throughput of public blockchains can be improved. It is important to give sufficient attention to how these issues can be eliminated to make blockchain a mainstream technology (Abou Jaoude and Saade, 2019). New data quality regulation models also need to be developed for data governance to generate a suitable transformation and acceptability of their operational services, procedures, and applications concerning blockchain technology. Due to the insufficient data governance regulations in blockchain technology, in addition to the lack of involvement of trusted parties, it becomes difficult for the legal authorities to provide proper access underlining other data quality concerns, such as security and privacy (Angraal et al., 2017; Liang et al., 2017; Kumar et al., 2018; Sharma, 2018; Zhang et al., 2018; Abou Jaoude and Saade, 2019; Agbo et al., 2019; Alonso et al., 2019; Beinke et al., 2019; Casino et al., 2019; Clim et al., 2019; Esmaeilzadeh and Mirzaei, 2019; Khezr et al., 2019; McGhin et al., 2019; Prokofieva and Miah, 2019; Shuaib et al., 2019; Siyal et al., 2019; Yaqoob et al., 2019; Khan et al., 2020; Maesa and Mori, 2020; Pandey and Litoriya, 2020; Adere, 2022; Attaran, 2022; Saeed et al., 2022). In addition, the key requirement in the blockchain is developing new data quality standards, as the lack of blockchain standards serves as another key challenge for communication because of insufficient interoperability (Tama et al., 2017; Beinke et al., 2019; Cao et al., 2019; Abu-Elezz et al., 2020; Tanwar et al., 2020; Yaqoob et al., 2021; Attaran, 2022). It is essential to formulate blockchain policies and governing standards to allow blockchain technology to be widely adopted in healthcare industries. In general, standards should include all goals established by official bodies. Furthermore, they should be flexible so that they can be modified based on the lessons learned and by a rapidly changing technological and global environment.

## Conclusion

Blockchain is a decentralized, transactional database technology that enhances business processes and constructs trustworthy data sharing among the chain. Therefore, this new technology enables users to verify, preserve, and synchronize the contents of a data sheet (a transaction ledger) replicated by multiple users. The research aims to pinpoint the gaps related to healthcare data quality challenges domain and provide future research directions for the use of blockchain. The results of this review study also aim to support professionals, practitioners, and stakeholders who wish to implement and manage transformation projects related to blockchain in healthcare sector. Moreover, increasing their understanding of the implied factors would be beneficial for the decision-making processes of their organizations. Blockchain is a new technology, and interest in academic studies has increased in recent years. The process of acceptance and implementation of new technology is complex within organizations. In addition, a scarcity of knowledge causes inappropriate decisions in managing projects and work routines. Subsequently, those decisive factors should be taken into consideration in the development and implementation process for the healthcare sector. As this technology is still in the early stages, there are possibilities for future research areas to present usability and the perceptions of users for the implementation of blockchain technology. The regulations and new business models should be updated based on technology requirements along with environmental, sustainability, economic, technological, and information management perspectives. High-quality academic studies should concentrate on additional empirically tested studies in healthcare sector.

## Author contributions

AA wrote the manuscript and was involved in all the revisions and writing of the manuscript, helped with explaining the data, and helped with the tables. OA acquired and analyzed all data for the study, assisted in writing the manuscript, and was involved in revisions. All authors contributed to the article and approved the submitted version.
